# A Review: The Styrene Metabolizing Cascade of Side-Chain Oxygenation as Biotechnological Basis to Gain Various Valuable Compounds

**DOI:** 10.3389/fmicb.2018.00490

**Published:** 2018-03-22

**Authors:** Michel Oelschlägel, Juliane Zimmerling, Dirk Tischler

**Affiliations:** ^1^Environmental Microbiology Group, Institute of Biosciences, Technische Universität Bergakademie Freiberg, Freiberg, Germany; ^2^Microbial Biotechnology, Ruhr University Bochum, Bochum, Germany

**Keywords:** cofactor regeneration, intrinsic cofactor usage, enantioselective biocatalysis, styrene metabolic pathways, whole cell biotransformation, oxidoreductases

## Abstract

Styrene is one of the most produced and processed chemicals worldwide and is released into the environment during widespread processing. But, it is also produced from plants and microorganisms. The natural occurrence of styrene led to several microbiological strategies to form and also to degrade styrene. One pathway designated as side-chain oxygenation has been reported as a specific route for the styrene degradation among microorganisms. It comprises the following enzymes: styrene monooxygenase (SMO; NADH-consuming and FAD-dependent, two-component system), styrene oxide isomerase (SOI; cofactor independent, membrane-bound protein) and phenylacetaldehyde dehydrogenase (PAD; NAD^+^-consuming) and allows an intrinsic cofactor regeneration. This specific way harbors a high potential for biotechnological use. Based on the enzymatic steps involved in this degradation route, important reactions can be realized from a large number of substrates which gain access to different interesting precursors for further applications. Furthermore, stereochemical transformations are possible, offering chiral products at high enantiomeric excess. This review provides an actual view on the microbiological styrene degradation followed by a detailed discussion on the enzymes of the side-chain oxygenation. Furthermore, the potential of the single enzyme reactions as well as the respective multi-step syntheses using the complete enzyme cascade are discussed in order to gain styrene oxides, phenylacetaldehydes, or phenylacetic acids (e.g., ibuprofen). Altered routes combining these putative biocatalysts with other enzymes are additionally described. Thus, the substrates spectrum can be enhanced and additional products as phenylethanols or phenylethylamines are reachable. Finally, additional enzymes with similar activities toward styrene and its metabolic intermediates are shown in order to modify the cascade described above or to use these enzyme independently for biotechnological application.

## Introduction

Styrene is an aromatic compound which occurs naturally in plants, fruits or nuts (Shirai and Hisatsuka, 1979; Warhurst and Fewson, 1994). Nevertheless, significant amounts of this toxic compound are released into the environment by industrial processes. This is reasoned by its importance for the synthesis of polyesters and plastics (Khaksar and Ghazi-Khansari, 2009). Remarkably, no significant styrene accumulation has been observed in the environment indicating the presence for natural degradation routes for this compound (Tischler, 2015). One important route to degrade styrene in nature is based on microbiological activity (Tischler, 2015). The corresponding pathways in such microorganisms are also important for the degradation of industrially released styrene and chemical analogous compounds, respectively. Furthermore, these metabolic routes harbor also a high potential for biotechnological purposes because enzymes, which are involved in these degradation pathways, offer the possibility for interesting reactions in order to gain a number of valuable compounds.

This review elucidates the most important degradations pathways and discussed the biotechnological potential of the route designated as side-chain oxygenation. The enzymes of this pathways as well as the pathway itself allow the production of styrene oxides, phenylacetaldehydes or phenylacetic acids (Tischler, 2015). These fine chemicals play an important role for various industries and are used to produce pharmaceutical, agricultural, or cosmetic products as well as flavors. Chiral styrene oxides, for example, are especially used for the synthesis of enantiomeric substances and pharmaceuticals (Badone and Guzzi, 1994; Hattori et al., 1995). Phenylacetaldehydes are used as fragrances in the perfume industry as well as for the synthesis of flavors, different pharmaceuticals, insecticides, fungicides, or herbicides (Hölderich and Barsnick, 2001). Phenylacetic acids are used as precursors for pharmaceutical products or drugs (Skoutakis et al., 1988; Milne et al., 2011; Zhu et al., 2011), e.g., for the production of penicillin (Douma et al., 2012) or receptor agonists and antagonists (Ghorai et al., 2008). Another example of a valuable phenylacetic acid is 4-isobutyl-α-methylphenylacetic acid which is better known as ibuprofen (Chen et al., 1999). Phenylacetic acids are also applied in fragrances or flavors and play an important role for the cosmetic or food industry (Fahlbusch et al., 2012). Modifications of the side-chain oxygenation to enlarge the substrate and product spectrum, e.g., to valuable products like 2-phenylethanols – an important disinfectant, flavor and precursor (Fahlbusch et al., 2012) – or 2-phenylethylamines, are also discussed. Furthermore, additional enzymes interacting with styrene and similar compounds are described.

## Styrene Pathways are Widespread in Numerous Microorganisms

**FIGURE 1:**
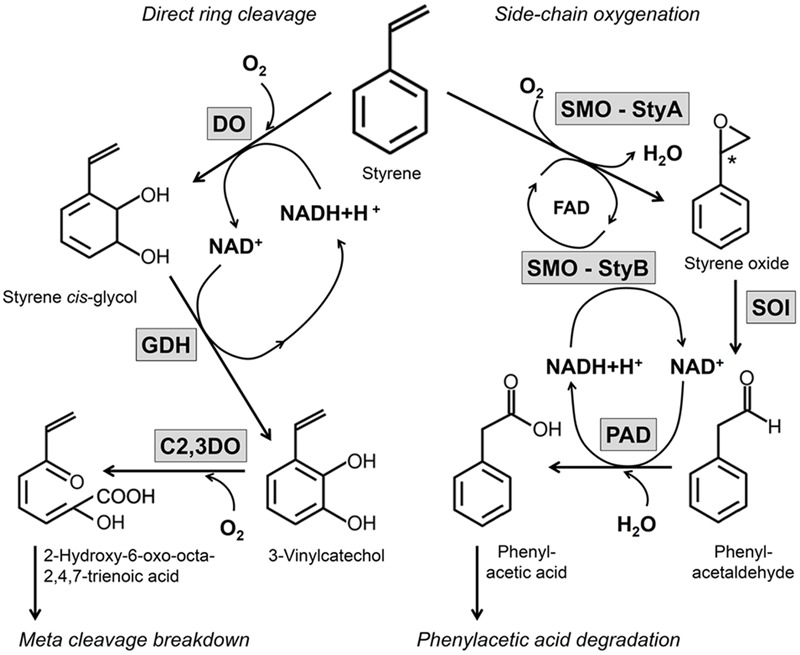
Overview on two important aerobic degradation routes of styrene in microorganisms. The aerobic degradation of side-chain oxygenation and the direct ring cleavage are illustrated in dependence of the enzymes involved and the cofactors needed. These both ways have been completely revealed in several microorganisms, so far.

One important way of natural styrene degradation is contributed to microorganisms and various studies have described the potential of different cultures and strains to degrade this aromatic compound (Baggi et al., 1983; Bestetti et al., 1984; Grbić-Galić et al., 1990; Warhurst et al., 1994; O’Connor et al., 1995; Itoh et al., 1996; Alexandrino et al., 2001; Lu et al., 2001; Patrauchan et al., 2008; Oelschlägel et al., 2012, 2014b; Toda and Itoh, 2012). Different ways of activating and metabolizing styrene by microorganisms are known. Styrene can be degraded under anaerobic conditions, but a clear mode of action or involved enzymes are not known yet (Tischler, 2015). Under aerobic conditions, two routes have been completely revealed in several microorganisms, so far. These ways are designated as direct ring cleavage and side-chain oxygenation (reviewed by O’Leary et al., 2002; Mooney et al., 2006; **Figure 1**).

Direct ring cleavage is combined with an initial dihydroxylation of the aromatic ring catalyzed by a 2,3-dioxygenase followed by a 2,3-dihydrodiol dehydrogenase (Warhurst et al., 1994; Patrauchan et al., 2008). These steps catalyze the oxidation of styrene to styrene *cis*-glycol followed by the formation of 3-vinylcatechol (**Figure 1**). This metabolite can be degraded into further metabolites by *ortho*- or *meta*-cleavage leading to the formation of central intermediates like acrylic acid, acetaldehyde, and pyruvate. This pathway is also known for the degradation of different aromatic compounds as ethylbenzene, toluene, phenol among others (Warhurst et al., 1994; Patrauchan et al., 2008) and represents an unspecific route for styrene (Tischler and Kaschabek, 2012; Tischler, 2015).

A second pathway, which has been described by several studies, attacks the vinyl-side chain of styrene (**Figure 1**). This route is designated as side-chain oxygenation and comprises a styrene monooxygenase (SMO), styrene oxide isomerase (SOI), and phenylacetaldehyde dehydrogenase (PAD) (O’Connor et al., 1995; Beltrametti et al., 1997; Itoh et al., 1997; Bestetti et al., 2004). The pathway of side-chain oxygenation seems to be a specific pathway for the degradation of styrene (Tischler, 2015) and was described for a number of organisms, e.g., *Corynebacterium* sp. AC-5 (Itoh et al., 1996), *Xanthobacter* sp. strain 124X (Hartmans et al., 1989), *Rhodococcus* sp. ST-5 (Toda and Itoh, 2012), *Rhodococcus opacus* 1CP (Tischler et al., 2009; Oelschlägel et al., 2012), *Sphingopyxis fribergensis* Kp5.2 (Oelschlägel et al., 2014b), and several *Pseudomonas* strains (O’Connor et al., 1995; Marconi et al., 1996; Beltrametti et al., 1997; Panke et al., 1998; Velasco et al., 1998; Bestetti et al., 2004; Park et al., 2006). This way efficiently transforms styrene into phenylacetic acid as central metabolite. Phenylacetic acid is an important metabolite for several pathways and undergoes degradation during a subsequent catabolic route (Olivera et al., 1998; Navarro-Llorens et al., 2005; Teufel et al., 2010). The initial step of this phenylacetic acid degradation is linked to a phenylacetate-CoA ligase which activates the acid to phenylacetyl-CoA (Teufel et al., 2010). Afterward, an 1,2-epoxide is formed on the aromatic ring by a multi-component epoxidase followed by an isomerization and hydrolytic ring cleavage. Finally, this pathway leads to the formation of the central metabolites acetyl-CoA and succinyl-CoA (Teufel et al., 2010).

It had been assumed that the pathway of side-chain oxygenation is the dominantly distributed route to metabolize styrene (Mooney et al., 2006). But, a more recent study has lately shown that out of 87 styrene-utilizing strains just 14 seemed to degrade styrene via the pathway of side-chain oxygenation which indicates that the direct ring cleavage, alternative and/or even modified pathways are more dominant (Oelschlägel et al., 2014b).

## Side-Chain Oxygenation as a Natural Cascade With Intrinsic Cofactor Regeneration

The styrene-degrading cascade designated as side-chain oxygenation comprises some – from a biotechnological point of view – interesting reactions. The initial step of this pathway (**Figure 1**) is performed by a two-component SMO which consists of an oxygenase (StyA) and a reductase (StyB) (reviewed by O’Leary et al., 2002; Mooney et al., 2006; Montersino et al., 2011; Huijbers et al., 2014). The SMO uses molecular oxygen in order to oxidize the vinyl side-chain to an epoxide. The styrene oxide obtained undergoes a subsequent isomerization to phenylacetaldehyde by a styrene oxide isomerase (SOI, StyC). The last step is catalyzed by a phenylacetaldehyde dehydrogenase (PAD, StyD, FeaB) and leads to the formation of the central metabolite phenylacetic acid (O’Leary et al., 2002; Mooney et al., 2006). Details about the proposed mechanisms of the enzymes in order to realize these reactions are discussed below in Section “More Details on the Enzymes of Side-Chain Oxygenation and Their Potential for Cell-Free Single-Step Reactions.”

Modifications of this route are also possible. One example for a modified side-chain oxygenation, which also yields phenylacetic acid, has been reported in case of *Gordonia rubripertincta* CWB2 (Oelschlägel et al., 2015b). This strain yielded all intermediates of the side-chain oxygenation, but harbors no SOI activity. Therefore, a different enzyme (or cascade) might be involved which needs to be proven. Lacking SOI activity has also been reported for *Rhodococcus* sp. ST-10 by Toda and Itoh (2012). There, a chemical conversion of the epoxide into phenylacetaldehyde and acetophenone has been discussed, but does not necessarily explain the fast growth of those SOI-deficient bacteria on styrene. Thus, the nature of this likely altered pathway awaits further exploitation.

The genes encoding the relevant enzymes for the side-chain oxygenation are commonly located in a *styABCD* gene cluster (Beltrametti et al., 1997; Panke et al., 1998; Velasco et al., 1998; O’Leary et al., 2001; Toda and Itoh, 2012; Oelschlägel et al., 2014b; reviewed by O’Leary et al., 2002; Mooney et al., 2006). Such clusters have been described for pseudomonads and some rhodococci which are similar to each other. But, the regulatory genes *styS* and *styR*, which are known to be present in *Pseudomonas* strains (Panke et al., 1998; Velasco et al., 1998; O’Leary et al., 2001; Tischler and Kaschabek, 2012), have not been found in the *sty* cluster of some *Rhodococcus* strains (Toda and Itoh, 2012; Oelschlägel et al., 2014b). More recently, the styrene degradation of a *Sphingopyxis* strain has been revealed in detail indicating larger differences within the clustering of the relevant genes (Oelschlägel et al., 2014b). In this strain, the PAD-encoding gene is located upstream of the *styABC* cluster and neighbored to genes which are responsible for the phenylacetic acid degradation. Furthermore, a hypothetical histidine kinase sensor protein and a LysR transcriptional regulator have been supposed to be responsible for gene regulation while *styS* and *styR* have not been found in the respective cluster, too (Oelschlägel et al., 2014b).

Nevertheless, the inducing effects of styrene and its metabolites were investigated independently from the detailed mechanisms of *sty*-gene regulation (Hartmans et al., 1989; O’Connor et al., 1995; Oelschlägel et al., 2014b). During the recent investigation of seven styrene-degrading strains of the genera *Pseudomonas, Rhodococcus, Sphingopyxis, Sphingobium* and *Xanthobacter*, it has been shown that styrene oxide was the most efficient inducer in four strains while styrene yielded the highest enzyme activities in two strains (Oelschlägel et al., 2014b). Phenylacetaldehyde has been identified as the best inducer in only one case while phenylacetic acid, the central product of the side-chain oxygenation, served not as an important inducer for the corresponding *sty* genes (Oelschlägel et al., 2014b). O’Connor et al. (1995) have previously published similar results and revealed that styrene oxide and phenylacetaldehyde yield the highest SOI and PAD activities in *Pseudomonas putida* CA-3 while phenylacetic acid harbors only a low inducing effect. Further studies about the inducing effect of substituted styrenes or metabolites seems meaningful to understand the sensitivity of the promoter toward these classes of inducers. Nevertheless, the promoters of these *sty*-gene clusters investigated, so far, are commonly restricted to the substrate and the metabolites of the side-chain oxygenation (Hartmans et al., 1989; O’Connor et al., 1995; Oelschlägel et al., 2014a,b). This dependence of *sty* promoters on reactive and at higher concentrations inhibiting or even toxic compounds as well as the circumstance, that the detailed mechanisms of induction are not completely understood in some cases, limits the application of such promoters in biotechnology.

The near location and – in most cases – same regulation of the enzymes of the upper pathway is also useful with respect to intrinsic cofactor regeneration (see also **Figure 1**). The SMO is an FAD-dependent enzyme which needs NADH as cofactor (Montersino et al., 2011; Huijbers et al., 2014). NADH cannot be replaced by NADPH and thus a respective cofactor supply is necessary. While FAD is regenerated after the epoxidation from hydroxyl-FAD by releasing water, NADH is oxidized to NAD^+^. Afterward, NAD^+^ is reduced again to NADH by the PAD during the last step of this catalytic route (Hartmans et al., 1989). Because the isomerization of the SOI is independent of cofactors (Itoh et al., 1997; Oelschlägel et al., 2012; Tischler, 2015), the side-chain oxygenation is independent from an external cofactor recycling system and all cofactors are regenerated in this cascade by the enzymes involved.

## More Details on the Enzymes of Side-Chain Oxygenation and Their Potential for Cell-Free Single-Step Reactions

### Styrene Monooxygenase

The SMO and related enzymes are grouped together as class E flavoprotein monooxygenases (EC: oxygenase-1.14.14.11 and reductase-1.5.1.36) and have some similar properties (Montersino et al., 2011; Huijbers et al., 2014). All of these monooxygenases represent two-component systems in which a NADH:FAD oxidoreductase (designated as SMOB, StyB, IndB, StyA2B) delivers reduced FAD by NADH-consumption. The reduced FAD is transferred either directly or by diffusion to the monooxygenase subunit(s) (designated as SMOA, StyA, IndA, StyA1, StyA2B) (Otto et al., 2004; Kantz et al., 2005; Tischler et al., 2010; Ukaegbu et al., 2010; Morrison et al., 2013). In this subunit the reduced FAD binds on the active site and molecular oxygen can be activated to yield a (hydro)peroxy-FAD which is able to attack the substrate (Kantz and Gassner, 2011; Tischler et al., 2013). During the subsequent substrate oxygenation hydroxyl-FAD is formed. Afterward, the product is released and water is eliminated from the hydroxyl-FAD leading to the initially oxidized FAD. The reaction cycle described above can be repeated now. This indicates only NADH is consumed by SMOs, which needs to be recycled externally, and that only FAD is internally re-used or -cycled.

Most of the SMOs described, so far, belong actually to a styrene specific degradation pathway. As mentioned above, the enzymes are commonly encoded in a *styABC* or *styABCD* gene cluster (Tischler et al., 2012; Tischler, 2015). These enzymes are classified as E1 flavoprotein monooxygenases. Besides those, some E2 type flavoprotein monooxygenases are known. They were also described as SMOs because they perform basically the same reaction, but the natural substrate was only hypothesized based on the uncovered genetic and metabolic background (Tischler et al., 2012). Recently, the enzymes belonging to this group of E2 type flavoprotein monooxygenases have been described to initiate indole degradation (Lin et al., 2015; Sadauskas et al., 2017). Thus, these enzymes likely can operate as SMOs, but other target substrates are supposed to be the natural target of respective enzymes. Hence, these monooxygenases are not a part of natural styrene-degrading cascades, but of course can be used in artificial systems.

SMOs have been extensively reviewed from a mechanistic and biotechnological point of view (van Berkel et al., 2006; Montersino et al., 2011; Huijbers et al., 2014). Thus, only some important facts are selected and summarized herein. The reductase units accept only NADH to reduce FAD for catalysis while NADPH is not used (Heine et al., 2017a,b). Furthermore, FMN and riboflavin can be reduced by this subunits, too. The nature of their *N*-terminus defines the acceptance and activity of these reductases (Heine et al., 2017a,b). The reduced FAD is then used by the monooxygenase unit to perform the biotechnological relevant catalysis. It needs to be mentioned that neither reduced FMN nor reduced riboflavin are accepted by the monooxygenase (Montersino et al., 2011). Nevertheless, it would be an interesting topic for protein engineering in order to alter this property and to allow the utilization of cheaper and more stable flavin cofactors. However, the reduced FAD then allows the activation of molecular oxygen and enables substrate oxygenation as described above.

For the transformation, various substrates have been reported beside styrene and indole (**Table 1**). For epoxidation, a number of styrene-analogous compounds can be used (Montersino et al., 2011). But, also aliphatic alkenes serve as substrates (Toda et al., 2012a,b). Remarkably, these reactions are highly stereospecific and very high enantiomeric excesses (ee) are reached in most cases. This feature of SMOs offers the access to interesting building blocks for several applications. In the case of indole, a corresponding reaction product has not been verified until now, but it can be assumed that also an indole oxide is formed (Sadauskas et al., 2017). Nevertheless, this assumption need to be proven. However, it can be supposed that indole analogous compounds can be converted as well. Furthermore, all E-type monooxygenases perform sulfoxidation, but with a different degree of enantioselectivity (van Hellemond et al., 2007; Tischler et al., 2010; Paul et al., 2015; Riedel et al., 2015). The products obtained are chiral sulfoxides and no over-oxygenation has been reported, so far. With respect to sulfoxidation, the direct FAD reduction by means of BNAH was established which allowed to omit the FAD-reductants (Paul et al., 2015).

**Table 1:** Substrates of SMOs and related proteins.

Substrate	Enantioselectivity	Comment	Reference^∗^
**Chiral epoxidation reactions**			
Styrene	(*S*)	Model substrate	
Indene	(*1S,2R*)	Target for pharmaceutical industries as a precursor of indinavir	Hollmann et al., 2003; Lin et al., 2010
Styrene with substitutions at the aromatic ring	(*S*)	Activity often similar to the model substrate styrene	
Styrene with substitutions at the vinyl chain	(*S*)	Mutations in the active site can change enantioselectivity in case of bulky substitutions	Lin et al., 2012; Toda et al., 2012a
Heterocyclic compounds	(*S*)	Formation of pyridine-like epoxides	Lin et al., 2010; Toda et al., 2012a
Non-conjugated alkenes including allylbenzenes	(*S*)	Much lower activity or rate of biotransformation compared to aromatic, conjugated substrates	Lin et al., 2010, 2011; Toda et al., 2012a,b, 2014
Indole	n.d.	Product not determined yet, but it auto-catalytically forms indigo in presence of molecular oxygen	Toda et al., 2012b; Sadauskas et al., 2017
**Chiral sulfoxidation reactions**			
Aromatic sulfides (e.g., thioanisole) including derivatives with substitutions at the aromatic ring	(*R*), (*S*)	Thioanisole (methyl phenyl sulfide) is the model substrate Enantioselectivity depends on the type of enzyme selected	van Hellemond et al., 2007; Paul et al., 2015; Riedel et al., 2015

*n.d., not determined. ^∗^References have been summarized by Montersino et al. (2011), Huijbers et al. (2014), and Tischler (2015). Furthermore, a number of important reference are given in the table to clarify comments or more recent findings.*

All in all, SMOs are very promising candidates for biocatalysis. Thus, biotechnological applications were investigated during several studies (Panke et al., 2000, 2002; Hofstetter et al., 2004; Ruinatscha et al., 2009; Kuhn et al., 2012). Panke et al. (2000, 2002) have established a process using a single E1 SMO from *Pseudomonas* sp. VLB120 in order to gain significant amounts of (*S*)-styrene oxide. Finally, up to 388 g styrene oxide were obtained from a 30-L fed-batch conversion in a fermenter within 16 h using whole cells of a recombinant *Escherichia coli* strain and bis(2-ethylhexyl)phthalate as organic phase (Panke et al., 2002). The average product formation corresponded to an activity of 170 U per liter (Panke et al., 2002). Earlier studies have reported activities of up to 300 U per liter, but under the usage of a 2-L-scale process (Panke et al., 2000). During more recent studies of Kuhn et al. (2012), whole cells of a *styC* knock-out strain of *Pseudomonas* sp. VLB120 were also used as whole cell biocatalyst in comparison to the recombinant *E. coli* cells (Kuhn et al., 2012). The epoxidation activities were equal in both cases with up to 100 U/g_Cell_ _Dry_ _Weight_ during a short-term activity assay, but differ during the biotransformation in presence of an organic phase (Kuhn et al., 2012). Finally, the *Pseudomonas* strain needed – despite of its higher tolerance toward the epoxide – a more than twofold higher transformation time to gain a comparable product level (Kuhn et al., 2012). Remarkably, the authors revealed that larger amounts of styrene reduce the epoxidation rate in the *Pseudomonas* strain. This has been explained by physiological aspects in the cells which are linked to the solvent tolerance of the strain, e.g., efflux pump effects which reduces the intracellular styrene concentration or a higher consumption of reduction equivalents by the complete system ensuring the solvent tolerance (Kuhn et al., 2012). Both effects increase together with an increasing substrate concentration.

Nevertheless, small amounts of 2-phenylethanol have been reported using *E. coli* cells as whole cell biocatalyst (Panke et al., 2000; Panke et al., 2002). This ethanol formation is contributed to unspecific reactions within the cells and not (or only in a low extent) caused directly by the SMO itself because 2-phenylethanol formation has also been reported by another study using a recombinantly expressed SOI in *E. coli* in presence of styrene oxide without an SMO (Oelschlägel et al., 2015a). No by-product formation was proven using the *styC* knock-out strain of *Pseudomonas* sp. VLB120 (Kuhn et al., 2012). Nevertheless, Kuhn et al. (2012) strongly assumed by-product formation in *Pseudomonas* similar as in *E. coli*. It has been suggested that phenylacetaldehyde is formed non-specifically as by-product during the SMO-catalyzed turnover of styrene to styrene oxide in both cases. While the phenylacetaldehyde undergoes complete degradation in the *Pseudomonas* strain, unspecific aldehyde dehydrogenases reduce the aldehyde to 2-phenylethanol in *E. coli* (Kuhn et al., 2012).

The cell-free application of the E1 SMO from *Pseudomonas* sp. VLB120 has also been reported for substituted styrenes (Hofstetter et al., 2004). More than 50 mM product were reached after 10 h applying 2 g/L lyophilized StyA in presence of StyB, dodecane as organic phase and a formate/formate dehydrogenase system for NADH recycling (Hofstetter et al., 2004). By-product formation has been reported during a cell-free application, too. In the case of Hofstetter et al. (2004), the corresponding substituted 2-phenylethanols and phenylacetaldehydes of the substrates applied were detected as by-products. In dependence of the cofactor recycling system, also further by-products can occur. Ruinatscha et al. (2009) have described the cell-free application of an SMO combined with reductive electrochemical cofactor regeneration. During this study, the product yields of styrene oxide decreased for the benefit of by-products as acetophenone and phenylacetaldehyde (Ruinatscha et al., 2009). The reason of the by-product formation in the latter case has been explained with a radicalic semiquinone attack on the formed (*S*)-styrene oxide contributed by the electrochemical FAD reduction (Ruinatscha et al., 2009).

Despite the small amounts of by-product, which has been observed during different studies, all of these applications point out the significant biotechnological potential of SMOs for various processes.

### The Styrene Oxide Isomerase

The SOI (EC: 5.3.99.7 – intramolecular oxidoreductase) represents the second and commonly the most active enzyme of the side-chain oxidation route (O’Connor et al., 1995; Beltrametti et al., 1997; Panke et al., 1998; Bestetti et al., 2004; Oelschlägel et al., 2014b). The cofactor-independent enzyme is membrane-embedded (Oelschlägel et al., 2012, 2014b) and catalyzes the transformation of styrene oxide into phenylacetaldehyde without the occurrence of by-products (Hartmans et al., 1989; O’Connor et al., 1995; Beltrametti et al., 1997; Itoh et al., 1997; Panke et al., 1998; Bestetti et al., 2004; Oelschlägel et al., 2012, 2014b). The membrane localization stabilized the enzymes toward environmental influences and – for a short time – against temperatures up to 50°C (Oelschlägel et al., 2012). During recent studies, two subgroups of SOIs have been described with respect to the protein size. The enzymes of *Pseudomonas* and *Rhodococcus* strains have a size of 168-169 AA which corresponds to a molecular weight of 18 kDa (Hartmans et al., 1989; Beltrametti et al., 1997; Itoh et al., 1997; Panke et al., 1998; Velasco et al., 1998; Oelschlägel et al., 2012, 2014b; Toda and Itoh, 2012). In contrast, a novel SOI from *S. fribergensis* Kp5.2 showed larger differences compared to the SOIs mentioned above and reached a size of 186 AA corresponding to 20 kDa (Oelschlägel et al., 2014b).

**FIGURE 2:**
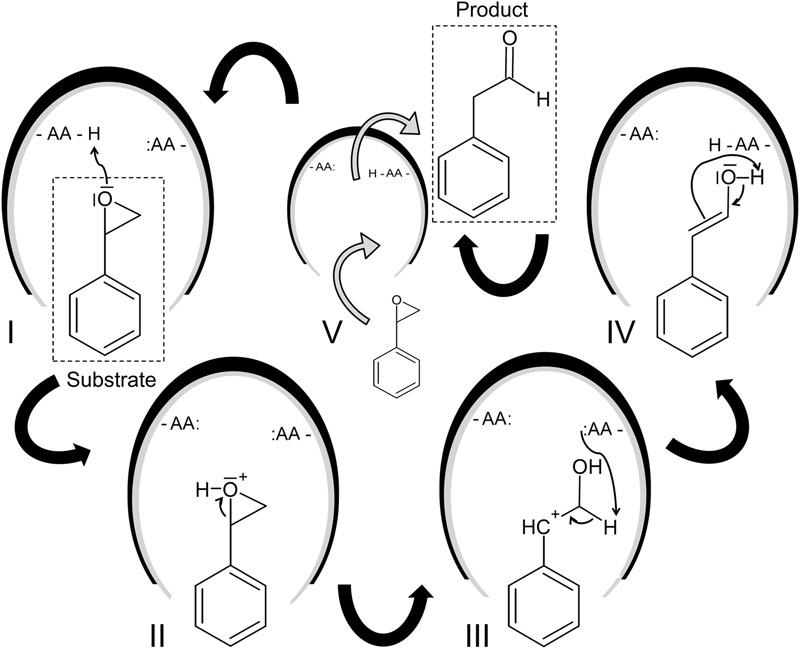
Proposed reaction mechanism of the SOI (Miyamoto et al., 2007). The putative mechanism of the SOI-catalyzed isomerization of styrene oxide into phenylacetaldehyde is illustrated based on the studies of Miyamoto et al. (2007), Oelschlägel et al. (2012), and Tischler (2015). The following steps are supposed: The initial protonation of the oxirane oxygen (**I** and **II**) causes ring opening during which a benzyl cation intermediate is formed (**III**). This reactive intermediate in transformed into an enol (**IV**) and undergoes keto-enol tautomerization yielding the aldehyde (V). The product is subsequently released and the reaction can start again.

Some previous studies have proposed a Meinwald rearrangement (Meinwald et al., 1963) as basic mechanism (Miyamoto et al., 2007; Oelschlägel et al., 2012) starting with the protonation of the oxirane oxygen which causes ring opening during which a benzyl cation intermediate is formed (**Figure 2**). This intermediate forms an enol and undergoes keto-enol tautomerization yielding the aldehyde. Recently, a second mechanism has been supposed which starts also with the formation of a benzyl cation intermediate, but this intermediate undergoes as stereoselective 1,2-hydrogen shift leading to the aldehyde (Wu et al., 2017a).

All SOIs showed a broad substrate tolerance for different ring-substituted styrene oxides (Itoh et al., 1997; Miyamoto et al., 2007; Oelschlägel et al., 2012, 2014b). Nevertheless, the SOI seems to be the bottleneck of the SMO-SOI-PAD cascade in the case of too bulky ring substituents as, for example, a 4-isobutyl-substitution (Oelschlägel et al., 2015b). Furthermore, a clear preference of the (*S*)-enantiomer of styrene oxide has been proven by several studies. Nevertheless, this privilege for a certain enantiomer seems to be substrate-depending and a slight preference for the (*R*)-enantiomer has been reported in the case of α-methylstyrene oxide (Wu et al., 2017a). The SOI-catalyzed isomerization is commonly associated with a loss of the chiral information introduced formerly by the SMO. Nevertheless, a recent study gives evidence for an enantioselective isomerization in the case of α-methylated substrates and an ee of up to 40% for the (*S*)-enantiomer was retained during (*S*)-4-chloro-α-methylstyrene oxide isomerization using the SOI from *Pseudomonas fluorescens* ST (Oelschlägel et al., 2015b). This has been proven by a more recent study with enantiopure α-methylstyrene oxide as substrate. Wu et al. (2017a) showed that the transformation of this substrate with an initial ee of 98% yielded a product with an ee of 91–95% using the enzyme from *Pseudomonas* sp. VLB120. It has been assumed that the SOI seems to retain a chiral information introduced commonly by the SMO and not introduce an own information directly during the isomerization process. Evidence for that was given by Wu et al. (2017a) using racemic α-methylstyrene oxide yielding a more or less racemic product. Nevertheless, the aspect that the SOI-catalyzed isomerization retains the stereochemical information is an important advantage toward the previously proposed chemical Meinwald rearrangement (Meinwald et al., 1963) because the latter one yields only a very low ee and the chiral information gets almost completely lost (Ertürk et al., 2010; Wu et al., 2017a).

The cofactor-independency and stability suggest this enzyme as a promising candidate for the biotechnological synthesis of phenylacetaldehydes from styrene oxides. Nevertheless, a product-mediated irreversible inhibition has been determined during several studies at high concentrations of about 50–80 mM for several SOIs, maybe contributed by covalent modification of the enzyme by the reactive phenylacetaldehyde (Oelschlägel et al., 2012, 2014b). Thus, different strategies were pursued to reduce this inhibiting effect. Some of these strategies dealt with the reaction step and tried to enhance the stability of the enzyme during the transformation. In this context it has been shown that a covalent immobilization of SOI on carriers together with the usage of these SOI-linked carries in a two-phase system with the non-toxic phthalate-related 1,2-cyclohexane dicarboxylic acid diisononyl ester reduced the susceptibility of the SOI and yielded 1.6–2.0-fold higher amounts of product (Oelschlägel et al., 2014a). Another way based on a selection of more stable SOIs. This way has been initiated because recent research supposed different susceptibilities of known SOIs toward aldehyde inhibition (Oelschlägel et al., 2014b). This hypothesis has been proven by a more recent study in which a significantly higher stability toward product inhibition was revealed for an enzyme from *S. fribergensis* Kp5.2 compared to SOIs from *Pseudomonas* and *Rhodococcus* strains (Oelschlägel et al., 2017). Finally, more than 300 mM phenylacetaldehyde (>36 g L^-1^) were obtained in this study within 60 min using this enzyme of strain Kp5.2. Remarkably, this is the highest product yield reported for this enzyme class, so far (Oelschlägel et al., 2017).

Another limiting factor using SOIs for several applications was - during a long time – the time-expensive production of the enzymes in the native hosts yielding – in dependence of the different organisms – activities of 3.1–21 U mg^-1^ after 7–28 days (Oelschlägel et al., 2014a,b). Despite the above mentioned membrane localization, the recombinant expression of codon optimized or native *styC* genes in *E. coli* lead to a significant optimization of the producing process. Remarkably, 6- to 7-fold higher activities have been described for the enzymes from *R. opacus* 1CP and *S. fribergensis* Kp5.2 compared to the activity in the native hosts and were generated within 1–2 days (Oelschlägel et al., 2017). Finally, this improved production together with the above mentioned strategies to reduce the product-mediated inhibition enhances significantly the applicability of this SOI – maybe also with respect to industrial processes.

### The Phenylacetaldehyde Dehydrogenase

The PAD (EC: 1.2.1.39) represents the third enzyme of the styrene specific side-chain oxygenation route (O’Connor et al., 1995; Beltrametti et al., 1997; Panke et al., 1998; Bestetti et al., 2004; Oelschlägel et al., 2014b). Nevertheless, PADs are not only involved in the degradation of styrene and are also important during the metabolization of 2-phenylethanol, ethylbenzene, phenylalanine or 2-phenylethylamine (Parrott et al., 1987; Hartmans et al., 1989; Shimizu et al., 1993; Corkery et al., 1994; Ferrández et al., 1997; Hanlon et al., 1997; Rodríguez-Zavala et al., 2006; Arias et al., 2008; Tischler, 2015). This is also the reason for a widespread occurrence of PAD genes in many organisms, but only a few studies are available about this enzyme class (Tischler, 2015). Until now, only a few microbial PADs have been investigated including these of *P. fluorescens* ST (Beltrametti et al., 1997), *Pseudomonas putida* S12 (Crabo et al., 2017), *E. coli* K-12 (Parrott et al., 1987; Ferrández et al., 1997; Hanlon et al., 1997; Zimmerling et al., 2017), *Arthrobacter globisformis* (Shimizu et al., 1993), *Xanthobacter* sp. 124X (Hartmans et al., 1989), *Rhodococcus rhodochrous* (Hartmans et al., 1990) and the yeast-like fungus *Exophiala jeanselmei* (Cox et al., 1996). Beltrametti et al. (1997) have revealed a strong homology between the StyD of *P. fluorescens* ST and other prokaryotic as well as even eukaryotic aldehyde dehydrogenases (ALDH, EC 1.2.1). The recently studied PAD of *P. putida* S12 was characterized as an *N*-terminal his-tagged protein and showed a structural similarity to sheep liver cytosolic aldehyde dehydrogenase (ALDH1) (Crabo et al., 2017). These facts confirm the ubiquitous appearance of PADs due to their important role in metabolisms (Liu et al., 1997).

Aldehyde dehydrogenases (ALDHs) are widespread over all life kingdoms (Perozich et al., 1999). Their active site containing of cysteine and glutamate is highly conserved and hence, the following catalytic mechanism is similar in all ALDHs (Hempel et al., 1993; Liu et al., 1997). The catalytic relevant cysteine binds temporarily on the carbon atom of aldehyde’s carbonyl group to release the hydride ion of this group which is transferred to NAD(P)^+^ (Liu et al., 1997; Crabo et al., 2017). Then, the essential water molecule is attacked by negatively charged glutamate and the remaining hydroxide ion binds on the carbon atom of the carbonyl group. In the last step, cysteine’s sulfur ion is unattached and deprotonated by glutamate’s acid group to form a thiol group. Finally, the aldehyde is converted into an acid, both amino acids are unaltered, one water molecule is consumed and the co-substrate is reduced to NAD(P)H (Crabo et al., 2017).

Despite that all ALDH share the same active site structures and catalytic mechanism, they differ in some properties like co-substrate preference, occurrence in certain pathways and, therefore, substrate specificity as well as catalyzed reactions (Perozich et al., 1999; Crabo et al., 2017). Thus, ALDH are divided into class I, II and III. Members of class I and II are homotetramers and catalytic dependent on metal ions like Mg^2+^, Ca^2+^ and Mn^2+^ (Takahashi and Weiner, 1980; Takahashi et al., 1980; Ho et al., 2005; Brichac et al., 2007). In contrast, the enzymes belonging to class III are homodimers and independent on metal ions (Crabo et al., 2017). The microbial PADs investigated, so far, belong to class III.

Focused on the above mentioned PADs, the substrate spectrum of these previously studied PAD enzymes is restricted on differently substituted phenylacetaldehydes like 4-chloro-, 4-fluoro, 4-hydroxy- und 3,4-dihydroxy phenylacetaldehyde – similar as described for SOIs (Ferrández et al., 1997; Hanlon et al., 1997; Rodríguez-Zavala et al., 2006; Zimmerling et al., 2017). Between these enzymes, different cofactor dependencies were revealed. The *E. coli* and fungus enzymes prefer strongly NAD^+^ (Ferrández et al., 1997; Rodríguez-Zavala et al., 2006; Zimmerling et al., 2017), whereas the PAD of *Arthrobacter globisformis* showed only 2% activity using NAD^+^ instead of NADP^+^ (Shimizu et al., 1993). Due to problems concerning the purity, availability and maximum applicable concentrations of the phenylacetaldehydes during the studies dealing with FeaB of *E. coli* K-12 (Hanlon et al., 1997; Ferrández et al., 1997), an enhanced assay has been reported which allows the *in-situ*-production of phenylacetaldehydes by using the well characterized and highly active SOI of *R. opacus* 1CP and styrene oxides as substrates (Zimmerling et al., 2017). This assay serves as distinguished approach for the characterization for phenylacetaldehyde metabolizing enzymes and opens new possibilities for further studies on PADs and related dehydrogenases.

From a biotechnological point of view, PADs seem to be promising candidates for the oxidation of aromatic aldehydes to corresponding acids (Tischler, 2015). First studies have revealed cell-free PAD activities of about 8.4–41 U mg^-1^ after recombinant expression and subsequent enrichment or purification (Hanlon et al., 1997; Zimmerling et al., 2017). The dependence on NAD^+^, NADP^+^ and/or phenazine methosulfate (PMS) allows the cell-free application of the enzyme only in presence of cofactor recycling systems (Hartmans et al., 1989, 1990; Corkery et al., 1994; Cox et al., 1996; Ferrández et al., 1997; Rodríguez-Zavala et al., 2006). Furthermore, phenylacetaldehyde and related substrates have been reported to inhibit the purified enzyme of strain K-12 already at concentration above 10 μM (Hanlon et al., 1997). This aspect needs an efficient solution to avoid too large aldehyde concentrations in presence of the enzyme - especially during cell-free applications. Thus, further studies investigating detailed characteristics as well as possible ways to realize an application are necessary to estimate the real potential of these enzymes for biocatalysis.

Beside the usage of the single enzymes from the side-chain oxygenation, the SMO, SOI and PAD can also be applied together in order to perform biotechnological multi-step syntheses. Such an application is discussed in the next chapter.

## The Biotechnological Potential of the Side-Chain Oxygenation Cascade

From a biotechnological point of view, the complete *sty* cascade comprising the enzymes mentioned in Section “More Details on the Enzymes of Side-Chain Oxygenation and Their Potential for Cell-Free Single-Step Reactions” can be used as promising way to gain phenylacetic acids from styrenes under intrinsic cofactor recycling. If the enzymes are applied in such a combination, it needs to be mentioned that the cascade is then limited to the rate-limiting SMO and therewith to the – to our knowledge – slowest enzyme of the cascade (Tischler, 2015). Nevertheless, the SMO activity can also be improved by a higher NADH-level and a total cascade turnover can be expected. Thus, an artificial high NADH level to start the reaction cycle seems to be beneficial in the case of a cell-free use. Another possibility for a biotechnological application offers a whole cell biocatalysis without the need to supply cofactors. Furthermore, a higher stability of the process can be expected in whole cells because reactive oxygen species, which can occur during the uncoupled reaction of the SMO subunits, are degraded in the cells. Therefore, this way of application seems to be the most reasonable way, so far.

The biotechnological aspect of a cascade comprising SMO, SOI and PAD has been investigated by a previous study in native organisms. While styrene is completely metabolized during this pathway, a co-metabolic transformation of substituted styrenes into phenylacetic acids has been reported (Oelschlägel et al., 2015b). The substituted co-products obtained undergo only a slow or, as reported for halogenated phenylacetic acids, no further metabolization by enzymes of the phenylacetic acid degradation pathway and are accumulated in the culture medium. This co-metabolic production strategy has been proven in *R. opacus* 1CP, *P. fluorescens* ST, *S. fribergensis* Kp5.2, and *G. rubripertincta* CWB2, so far (Oelschlägel et al., 2015b). Remarkably, the substrate tolerance and transformation yields differ significantly between the strains and *P. fluorescens* ST has been reported to be the fastest candidate for the production of 4-chloro-, 4-fluoro-, 3-chloro-, α-methyl- and 4-chloro-α-methylphenylacetic acid (Oelschlägel et al., 2015b). Initial experiments during this study with strain ST yielded about 28 mM 4-chlorophenylacetic acid after nearly 350 days (Oelschlägel et al., 2015b). Furthermore, the transformation of chiral products, as 4-chloro-α-methylstyrene, has been reported during this study. In some cases, a stereoselective preference has been shown and an ee of 40% for (*S*)-4-chloro-α-methylphenylacetic acid has been reported. The same study has shown that substrates with bulkier substitutions, e.g., in the case of 4-isobutyl-α-methylstyrene, were not transformed into the corresponding 4-isobutyl-α-methylphenylacetic acid – better known as ibuprofen – by the native cascade of side-chain oxygenation (Oelschlägel et al., 2015b). This is probably caused by an exhausted substrate spectrum of the SOI, because *G. rubripertincta* CWB2, which harbors a modified pathway of this cascade and in which the isomerase is likely substituted by other enzymes, is also able to transform the isobutyl-substituted substrate into ibuprofen (Oelschlägel et al., 2015b). In all cases, the co-metabolism is quite slow and further optimization is needed for a biotechnological approach. Nevertheless, this way offers the opportunity for the production of such important products without any genetic modifications of microorganisms.

Beside the native cascade, recombinant biocatalysts based on *E. coli* have been investigated in recent studies using selected enzymes from native hosts. This was also initiated in order to produce phenylacetic acid from styrene without – in contrast to the application of native styrene degraders – further product metabolization. Initial studies investigated the transformation of styrene oxide by an SOI in *E. coli* BL21(DE3) in order to produce phenylacetaldehyde from styrene oxide (Oelschlägel et al., 2015a). Because *E. coli* harbors an own PAD (FeaB), the modification of this *E. coli* strain by introducing a single SOI gene already lead to the formation of phenylacetic acid as main product (Oelschlägel et al., 2015a). Phenylacetaldehyde occurs only as an intermediate and undergoes further oxidation by FeaB during these experiments. A further gene-knock-out has been discussed to enhance the yield of phenylacetaldehyde (Oelschlägel et al., 2015a). Nevertheless, phenylacetaldehyde represents also a toxic compound for the *E. coli* cells at concentrations of 1.35 mM which limits this way of application (Oelschlägel et al., 2015a). Thus, an improvement of the recombinant phenylacetic acid production has been evaluated as more meaningful and a further linkage with an SMO was intended to enlarge the substrate spectrum to styrenes, too (Oelschlägel et al., 2015a). This has been realized by a novel study of Wu et al. (2017b). The combination of an SMO and SOI from *Pseudomonas* sp. VLB120 and an *E. coli* PAD in an *E. coli* expression host and its application in a two-phase system with ethyl oleate yielded 122 mM phenylacetic acid from 130 mM styrene during 6 h. Additionally, various ring-substituted styrenes containing F-, Cl-, Br-, methyl-, and methoxy-substituents were transformed successfully to the corresponding products with yields of 85 to >99% from initially 25 or 50 mM of substrate (Wu et al., 2017b). Remarkably, stereoselective transformations have also been reported and (*S*)-2-phenylpropanoic acid was obtained from α-methylstyrene with an ee of 88%, respectively (Wu et al., 2017b).

## Variants Combining Smo and Soi With Further Enzymes During Whole Cell Biocatalyses

The cascade of side-chain oxygenation, which is discussed in Section “The Biotechnological Potential of the Side-Chain Oxygenation Cascade,” offers a basis for further modification in order to shift the transformation into various directions gaining higher yields or other products as well as to use other substrates for the catalysis. Such modifications are described below.

One modification is based on the last enzyme of the cascade and allows an optimization of the enantiomeric excess during the production of chiral phenylacetic acids. This modification based on findings of Könst et al. (2012) by screening a c-LEcta collection of alcohol dehydrogenases with the substrate 2-phenylpropionaldehyde. 13% of these alcohol dehydrogenases were able to transform this aldehyde also into the corresponding carboxylic acid beside their alcohol-oxidizing activity (Könst et al., 2012). Nevertheless, this acid formation has been reported to depend strongly on the pH (Könst et al., 2012). During the same study, one enzyme was subsequently selected and protein-engineered to improve the activity and selectivity, respectively. The modified enzyme has been designated as ADH-9V1. Finally, this enzyme allows a highly efficient production of the acid toward the alcohol if an efficient NAD^+^ regeneration system is present (Könst et al., 2012). Wu et al. (2017b) used ADH-9V1 instead of a PAD in a cascade with SMO and SOI, too. This substitution leads also to the formation of phenylacetic acids, but improves significantly the ee of chiral products compared to the native cascade. In the case of α-methylstyrene, the ee for (*S*)-2-phenylpropanoic acid was improved from initially 88% (with PAD from *E. coli*) to 98-99% (with ADH-9V1). An ee value of 92–98% has also been reported for *p*-Cl-, *p*-F- and *p*-methyl-substituted α-methylstyrenes (Wu et al., 2017b).

The substitution of PAD with an aldehyde reductase or a common alcohol dehydrogenase without acid-forming activity leads to the formation of alcohols from styrenes. Wu et al. (2017a) combined a phenylacetaldehyde reductase (PAR) from *Solanum lycopersicum* with the SMO and SOI from *Pseudomonas* sp. VLB120 in an *E. coli* expression host and applied the cells in a two-phase system using hexadecane as the organic phase. 60 mM of styrene were transformed into 2-phenylethanol reaching yields of 93% after 8 h (Wu et al., 2017a). Beside styrene, also several other ring-halogenated, ring-methylated as well as ring-methoxylated substrates were successfully transformed with yields of 60 to >99% (Wu et al., 2017a). During this study also the transformation of the pro-chiral α-methylstyrene was investigated yielding an ee of 87% of the respective (*S*)-product. Furthermore, Wu et al. (2017a) offer an opportunity to improve this ee to 97% using horse liver alcohol dehydrogenase instead of PAR.

Styrene can also be transformed into 2-phenylethylamine by slight modification of the above mentioned cascade. Substitution of PAD by a transaminase from *Chromobacterium violaceum* and *L*-alanine dehydrogenase from *Bacillus subtilis* together with an SMO and SOI from strain VLB120 in a modified *E. coli* strain (deletion mutant for disturbing dehydrogenases) allowed the transformation of 80 mM styrene into 2-phenylethylamine with yields of 93% within 10 h (Wu et al., 2017a). As mentioned for the 2-phenylethanols above, also substituted substrates were successfully transformed. In the case of chiral products as α-methyl-2-phenylethylamine, the authors offer also possibilities to enhance the ee to finally >90% for the corresponding (*S*)-enantiomer.

Beside these modifications described above in order to change to products of this cascade, also the spectrum of acceptable substrates can be enlarged by introducing additional enzymes. McKenna and Nielsen (2011) have reported the transformation of glucose into styrene. The combination of a phenylalanine ammonia lyase from *Arabidopsis thaliana* and *trans*-cinnamate decarboxylase from *Saccharomyces cerevisiae* in an *E. coli* strain leads to the transformation of *L*-phenylalanine to styrene (McKenna and Nielsen, 2011). *L*-phenylalanine is formed from glucose during the natural glycolysis and shikimate pathway in *E. coli*. A similar strategy comprises tyrosine ammonia lyase (from *Rhodotorula glutinis*) and *p*-hydroxycinnamic acid decarboxylase (from *Lactobacillus plantarum*) in *E. coli* which allows the transformation of the glucose-intermediate tyrosine into *p*-hydroxystyrene (Qi et al., 2007). Because of the toxicity of styrene for *E. coli* hosts, an improved production of *p*-hydroxystyrene has been reported using the solvent-tolerant *P. putida* S12 harboring the tyrosine ammonia lyase from *Rhodosporidium toruloides* and the decarboxylase mentioned above (Verhoef et al., 2009). Application of this strain in a two-phase system with 1-decanol resulted in nearly 150 mM of product. The combination of these enzymes, which allow the transformation of glucose into styrene and *p*-hydroxystyrene, with the enzyme cascades mentioned above should allow the formation of the corresponding phenylacetic acids, 2-phenylethanols and 2-phenylethylamines starting from glucose as a non-petrochemical compound.

Additionally, cinnamic acids offer natural and renewable substrates for the cascades discussed above because these acids are important ingredients in several plants. Cinnamic acids can be transformed into the corresponding styrenes by suitable phenolic acid decarboxylases. Nevertheless, the substrate spectrum of these decarboxylases is commonly limited to only a few substrates. The *p*-coumaric acid and caffeic acid, for example, serve as substrate for *p*-coumaric acid decarboxylase from *Lactobacillus plantarum* while no ferulic acid is transformed (Cavin et al., 1997a,b). In contrast, another ferulate and *p*-coumarate decarboxylase from *Bacillus pumilus* catalyzes only reactions with *p*-coumaric acid and ferulic acid (Degrassi et al., 1995). The phenolic acid decarboxylase from *Bacillus subtilis* has a slightly enhanced substrate spectrum and is able to transform caffeic acid, ferulic acid and *p*-coumaric acid, respectively (Cavin et al., 1998). Another protein from *Saccharomyces cerevisia* designated as PAD1 is able to convert *trans*-cinnamic acid (Richard et al., 2015). If this PAD1 is expressed together with a further protein from *S. cerevisia*, which is designated as FDC1, also *p*-coumaric acid and ferulic acid serve as substrates (Richard et al., 2015). All of these enzymes can be easily produced in significant amounts by recombinant expression in *E. coli* (Degrassi et al., 1995; Cavin et al., 1997a, 1998; Richard et al., 2015). The consideration of these enzymes in the above-mentioned side-chain oxygenation cascade should allow the production of non- or differently hydroxylated and/or methoxylated phenylacetic acids, 2-phenylethylamines or 2-phenylethanols in dependence of the cinnamic acid initially provided to the system.

**FIGURE 3:**
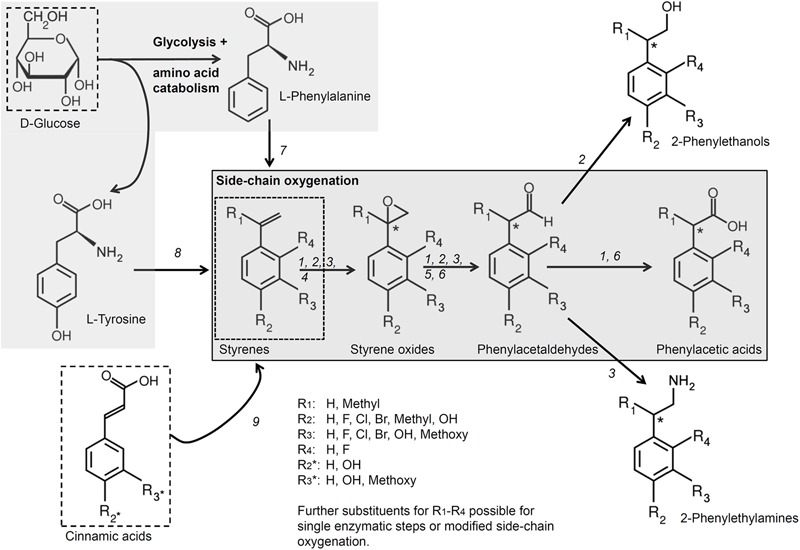
Important reactions and modifications based on the side-chain oxygenation. The side-chain oxygenation offers a basis for various reactions (illustrated by the numbers **1**–**9**). The native reaction from styrene to phenylacetic acid by SMO, SOI and PAD (**1**; Oelschlägel et al., 2015a,b; Wu et al., 2017b) can be modified by different ways: **2**: transformation of styrenes to 2-phenylethanols by using a aldehyde reductase or alcohol dehydrogenase instead of PAD (Wu et al., 2017a); **3**: transformation of styrenes to 2-phenylethylamines by usage of a transaminase and *L*-alanine dehydrogenase instead of PAD together with host modification (Wu et al., 2017a). Also single reactions as epoxidation (**4**; e.g., Panke et al., 1998 or reviewed by Montersino et al., 2011), isomerization (**5**; Oelschlägel et al., 2017), and aldehyde oxidation (**6**; Hartmans et al., 1989; Crabo et al., 2017; Zimmerling et al., 2017) can be performed. The usage of other substrates is also possible: **7**: usage of glucose to yield styrene by additional usage of a phenylalanine ammonia lyase and *trans*-cinnamate decarboxylase (McKenna and Nielsen, 2011) or **8**: to yield *p*-hydroxystyrene by tyrosine ammonia lyase and *p*-hydroxycinnamic acid decarboxylase (Qi et al., 2007; Verhoef et al., 2009); **9**: usage of cinnamic acids to gain styrenes by additional usage of different decarboxylases (Degrassi et al., 1995; Cavin et al., 1997a,b, 1998; Richard et al., 2015). Details are given in the Section “The Biotechnological Potential of the Side-Chain Oxygenation Cascade” and “Variants Combining SMO and SOI With Further Enzymes during Whole Cell Biocatalyses.

All reactions based on the cascade of Section “The Biotechnological Potential of the Side-Chain Oxygenation Cascade” as well as all modifications of this cascade described in Section “Variants Combining SMO and SOI With Further Enzymes during Whole Cell Biocatalyses” are finally summarized in **Figure 3**. Details are also given in the figure description.

Especially for the SMO, some alternative enzymes with similar activities are known which can be used to replace the SMO in the cascade. Furthermore, other enzymes, which enable other reactions with styrene, can be used in a biotechnological context, either in the cascade mentioned above, in parts thereof, or as single enzymes, respectively. Such additional enzymes are discussed in the next chapter.

## Further Enzymes With Similar Activities Toward Styrene and Its Metabolic Intermediates

There are a number of enzymes described which can act on styrene or chemically analogous compounds. Some of these as the SMOs (described above), the styrene dioxygenases or the P450 systems have a physiological relevance as they are part of styrene degradation or detoxification pathways (Tischler, 2015). But, most of the here listed enzymes can convert styrene due to a certain substrate promiscuity which is often an advantage for the development of novel bioprocesses. Furthermore, there are probably more enzymes acting on styrene, but have either not been described or tested in that respect. Thus, the following overview summarizes only the actual state, but is likely to be expanded.

### Monooxygenases and Their Potential for Styrene Transformations

P450-monooxygenases (P450s) are enzymes catalyzing a number of interesting reactions and often accept a range of substrates (Lechner et al., 2015). This and the known details on structure and mechanism qualifies these enzymes as valuable biocatalysts (Hrycay and Bandiera, 2015). And also for this class of enzymes styrene and its derivatives serve as model substrates to compare substrate turnover, by-product formation as well as enantioselectivity (Lechner et al., 2015). Many P450s convert selectively styrene into styrene oxide (Fruetel et al., 1992, 1994; Cox et al., 1996; Li et al., 2001, 2013). In dependence of the enzyme / host organism various selectivities have been reported and it is possible to produce (*R*)- or (*S*)-styrene oxide (Lechner et al., 2015). Furthermore, it is possible to improve the enantioselectivity by mutagenesis. Due to the high oxygenation power of P450s and based on the mechanism, often by-products are observed (Hrycay and Bandiera, 2015; Lechner et al., 2015). Nevertheless, such by-product formations can be reduced by process development. During a biotechnological application these enzymes are commonly used as whole cell systems in order to provide sufficient amounts of reducing equivalents (Urlacher and Girhard, 2012).

Hydrogen peroxide is used by peroxygenases to oxygenate substrates as styrene and chemically analogous compounds (Kluge et al., 2012; Hrycay and Bandiera, 2015; Wang et al., 2017). The oxygen transfer mechanism onto the substrate is similar to heme-monooxygenases in spite of the different oxygen-source (Hrycay and Bandiera, 2015). The heme-thiolate peroxygenase from *Agrocybe aegerita* and related enzymes seem to be promising candidates for biocatalysis and allow the oxygenation of various substrates at certain enantioselectivities (Kluge et al., 2012; Lechner et al., 2015). Especially, (1*R*,2*S*)-*cis*-*β*-methylstyrene oxide is produced at high rates and up to >99% purity (Kluge et al., 2012; Zhang et al., 2016). Furthermore, the first structural and mutagenetic studies have been conducted (Piontek et al., 2013; Zhang et al., 2016). Based on these results, an increasing application of these enzymes for biocatalysis can be expected.

In analogy to many oxygenases also the xylene monooxygenase (XMO) is somewhat promiscuous to substrates and allows also the epoxidation of styrene and its derivatives (Wubbolts et al., 1994a,b; Panke et al., 1999). The enantioselectivity is not sufficient enough for biocatalysis. Nevertheless, the enzyme harbors potential to develop relevant systems and seems also to be interesting to develop cascades as discussed above.

### Dioxygenases Interacting With Styrenes

The degradation of styrene *via* unspecific pathways, which starts with a hydroxylation of the aromatic nucleus, has been reviewed several times (Warhurst and Fewson, 1994; O’Leary et al., 2002; Mooney et al., 2006; Tischler and Kaschabek, 2012; Tischler, 2015). The initial dihydroxylation is performed by styrene dioxygenases (SDO) and if the enzymes could be considered to act mainly on styrene, they can be designated as styrene dioxygenases (Warhurst et al., 1994). The dioxygenase uses NADH in order to deliver reducing equivalents for oxygen activation and releases NAD^+^ which needs to be recycled (Warhurst et al., 1994). In the natural pathway of aromatic compound degradation *via* ring attack, this is achieved by the subsequent activity of a dehydrogenase (DH). This DH reduces NAD^+^ to NADH and re-aromatizes the compound to yield the respective catechol (Warhurst et al., 1994). Finally, the combination of these two enzymes, the SDO and the DH, allows NADH-recycling (cofactor) for the catalysis. However, it should be mentioned that this enzyme system is really promiscuous with respect to the substrates converted and most likely the enzymes were not evolutionary evolved for styrene, but for another aromatic compound (Tischler and Kaschabek, 2012; Tischler, 2015). Nevertheless, this broad substrate range of respective dioxygenases and dehydrogenases is useful in terms of application. This allows the production of various catechol derivatives which are interesting building blocks or chelating agents (Warhurst et al., 1994).

Whole cells of some *Pseudomonas* strains allow to dioxygenate the aromatic nucleus or vinyl side-chain of 2-bromostyrene (Königsberger and Hudlicky, 1993). Details on the enzyme have not been investigated and reported, so far. The products obtained show a promising high enantiomeric purity with an ee of >91% (Königsberger and Hudlicky, 1993). Remarkably, strain *P. putida* 39/D converts 2-bromostyrene into (1*S*,2*R*)-4-bromo-3-ethenylcyclohexa-3,5-diene-1,2-diol and (1*R*)-1-(2-bromophenyl)ethan-1,2-diol (Königsberger and Hudlicky, 1993). Both products indicate that an attack on the ring as well as on the side-chain occurs. Compared to that, the *Pseudomonas* strain NCIB 9816-11 does only attack at the vinyl side-chain and thus yields (1*R*)-1-(2-bromophenyl)ethan-1,2-diol (Königsberger and Hudlicky, 1993).

The naphthalene dioxygenase is a multicomponent enzyme which catalyzes the dioxygenation of naphthalene to yield the corresponding dihydrodiol (Resnick et al., 1996). As it activates molecular oxygen and transfers two oxygen atoms onto the substrate, it can provide access to valuable diols. Styrene was tested as a model compound in order to get the corresponding diol at a high enantioselectivity (Lee and Gibson, 1996; Tischler, 2015). Interestingly, it has been shown that the NDO of a pseudomonad could attack the vinyl side-chain as proposed and yielded (*R*)-1-phenyl-1,2-ethandiol (about 78% ee) (Lee and Gibson, 1996). Other studies used SMO together with epoxide hydrolases to gain the same product (Breuer et al., 2004; Tischler, 2015). It need also to be mentioned that substrates as indene and dihydronaphthalene are also dihydroxylated by NDOs instead of being epoxidized as with SMOs (Lee and Gibson, 1996). Thus, this seems to be a complementary approach to get to a number of valuable building blocks.

### Further Enzymes Interacting With Styrene

Recently, Wuensch et al. (2013) have reported two activities of phenolic acid decarboxylases on *p*-hydroxyl styrene derivatives. In one case they found the enzymes could work as an enantioselective hydratase to form 1-(*p*-hydroxyphenyl) ethanol-like compounds (Wuensch et al., 2013). This reaction was dependent on the buffer and especially the concentration of bicarbonate. Both aspects enable to tune stereoselectivity as well as turnover. Later, it was found that the same phenolic acid decarboxylases can use bicarbonate as a carbon dioxide source in order to perform a *β*-carboxylation of substrates (Wuensch et al., 2015). In this case *p*-hydroxyl styrene was transformed into (*E*)-cinnamic acid. However, the degree of substitutions on the aromatic nucleus influences the transformation efficiency and can also prevent any conversion in some cases, especially at a high degree of substitutions (Wuensch et al., 2015). Respectively, the *p*-hydroxyl group was mandatory and could not be omitted (Wuensch et al., 2015).

Peroxidases are versatile catalysts and can use hydrogen peroxide or organic peroxides as *tert*-butyl hydroperoxide to form a reactive oxygen which gets transferred onto the substrate, as for example styrene (Ortiz de Montellano and Grab, 1987; Colonna et al., 1993; Santhanam and Dordick, 2002). Furthermore, styrene serves as a model substrate for these enzymes and epoxidations have been frequently reported for many peroxidases (Ortiz de Montellano and Grab, 1987; Colonna et al., 1993; Santhanam and Dordick, 2002). However, these enzymes have usually low selectivities and thus products with a low ee are obtained or by-products are formed. For example, the chloroperoxidase from *Caldariomyces fumago* allows the formation of (*R*)-styrene oxide, but only with an ee up to only 68% (Colonna et al., 1993). This low enantioselectivity in combination with low turnover numbers are unfavorable for an application of these enzyme, which even shows unproductive activity as catalase (Lechner et al., 2015). However, these enzymes allow enzymatic access to (*R*)-enantiomers whereas SMOs, so far, only produce (*S*)-enantiomers as described above.

Lipase-mediated styrene epoxidation has been shown to be a very promising alternative to produce respective epoxides (Zhou et al., 2017a). However, it need to be mentioned that the actual catalysis on styrene or other alkenes is achieved by the lipase produced peracid (Zhou et al., 2017a). Overall, the conversions are high, but they need a rather long reaction time up to 24 h (Zhou et al., 2017a). Styrene was employed as a model substrate among others, which shows the applicability of the system (Zhou et al., 2017a). Interestingly, the epoxidation of alkenes allows recycling of a substrate based on the reaction cascade as follows: The lipases (e.g., CALB) employs H_2_O_2_ and a carboxylic acid to form H_2_O and the peracid (Zhou et al., 2017a). Subsequently, the peracid spontaneously reacts with an alkene to form the desired product (here: an epoxide) and while doing so the carboxylic acid is also formed and released. This carboxylic acid can become again a lipase substrate. Nevertheless, this system is at the beginning of a promising route as other reports show the potential to drive this kind of catalysis under various conditions and to minimize by-products and waste streams (Ranganathan et al., 2016, 2017; Zhou et al., 2017b).

## Conclusion

The side-chain oxygenation offers a basis for different syntheses in order to gain valuable compounds as styrene oxides, phenylactaldehydes and phenylacetic acids from styrene and derivatives thereof (see “More Details on the Enzymes of Side-Chain Oxygenation and Their Potential for Cell-Free Single-Step Reactions” and “The Biotechnological Potential of the Side-Chain Oxygenation Cascade,” **Figure 3**). A modification of the enzyme cascade by its combination with other dehydrogenases and decarboxylases enhances the substrate spectrum to natural compounds as cinnamic acids or allowed the synthesis of further products like 2-phenylethanols or 2-phenylethylamines (see Variants Combining SMO and SOI With Further Enzymes during Whole Cell Biocatalyses, **Figure 3**). Furthermore, the enzymes described in Section “Further Enzymes With Similar Activities toward Styrene and Its Metabolic Intermediates” provide a basis for further applications combining them with enzymes from the *sty* cascade, modifications thereof, or at least using them as single biocatalyst interacting with styrene.

The application of these enzymes in a cell-free way or in whole cell biocatalysts offer promising and environmental-friendly alternatives to already existing chemical or even biotechnological ways in order to gain such valuable products. The large number of studies about these enzymes and their applications give reasons to expect future improvements and probably a breakthrough into industrial processes.

## Author Contributions

The authors (MO, JZ, and DT) contributed equally to the presented review, drafted the manuscript together and approved the final version.

## Conflict of Interest Statement

The authors declare that the research was conducted in the absence of any commercial or financial relationships that could be construed as a potential conflict of interest.
